# Association between diet quality and obesity indicators among the working-age adults in Inner Mongolia, Northern China: a cross-sectional study

**DOI:** 10.1186/s12889-020-09281-5

**Published:** 2020-07-25

**Authors:** Lu Jia, Haiwen Lu, Jing Wu, Xuemei Wang, Wenrui Wang, Maolin Du, Peiyu Wang, Sha Du, Yuenan Su, Nan Zhang

**Affiliations:** 1 Department of Health Statistics, School of Public Health, Inner Mongolia Medical University, Hohhot, 010110 China; 2 Department of Medical Imaging, Affiliated Hospital of Inner Mongolia Medical University, Inner Mongolia Medical University, Hohhot, 010050 China; 3 National Center for Chronic and Non-Communicable Disease Control and Prevention, Chinese Center for Disease Control and Prevention, Beijing, 100050 China; 4 Department of Chronic Disease Control and Prevention, Inner Mongolia Center for Disease Control and Prevention, Hohhot, 010031 China; 5 Department of Social Medicine and Health Education, School of Public Health, Peking University Health Science Center, Beijing, 100191 China; 6 Department of Hygienic Toxicology, School of Public Health, Inner Mongolia Medical University, Hohhot, 010110 China

**Keywords:** Diet quality, Obesity, Working-age population, DASH diet, Mediterranean diet

## Abstract

**Background:**

Obesity is a major risk factor for the global burden of disease in countries that are economically developed or not. This study aimed to investigate the association between diet quality and obesity indicators applying DASH and aMed.

**Methods:**

This cross-sectional study on adult nutrition and chronic disease in Inner Mongolia (*n* = 1320). Dietary data were collected using 24-h diet recall for 3 consecutive days and weighing method. DASH and aMed were used to assess the dietary quality. WC, BMI and WC-BMI were used as obesity indicators. Logistic regression models were used to examine the associations between diet quality and obesity indicators.

**Results:**

Higher diet quality, assessed by DASH, was only associated with WC. The odds ratio (*OR*) for abdominal obesity in the highest tertile of DASH scores compared with the lowest was 0.71 (95% confidence interval (*CI*) 0.53, 0.96; *P*_trend_ = 0.03). Furthermore, aMed was inversely associated with obesity indicators. OR for abdominal obesity in the highest tertile of aMed score compared with the lowest were 0.63 (95% CI 0.47, 0.87; *P*_trend_ = 0.005) and 0.57 (95% *CI* 0.41, 0.77; *P*_trend_ = 0.02) for overweight and obesity, respectively, and 0.60 (95% *CI* 0.44, 0.81; *P*_trend_ = 0.02) for high obesity risk.

**Conclusions:**

Our findings suggest that dietary quality assessed using aMed is more closely associated with obesity than assessment using DASH in working-age adults in Inner Mongolia. The Mediterranean diet can be recommended as a healthy diet to control weight.

## Background

Obesity is a worldwide public health problem, with increased risks of cardiovascular and cerebrovascular diseases, diabetes, and probability of death [1]. Body mass index (BMI) and waist circumference (WC) are common obesity indicators. BMI is more closely related to general obesity, and WC is more closely related to abdominal obesity and metabolic related diseases [2]. In 2017 Global Burden of Disease (GBD) study [3], high body mass index (BMI) was ranked fourth among mortality risk factors. As obesity indicator, BMI-WC was a weight-related risk outcome combined WC and BMI. A Canadian study examined the association between occupational sedentary behavior and BMI-WC, and found that those at very high risk and extremely high risk were associated with increasing occupational sedentary time compared to the lowest obesity risk population among men [4]. Great changes have taken place in the spectrum of disease as well as the age and occupational structure of the patient population in China over the past few decades [5]. The working-age adults are at high risk for obesity, and the prevalence of overweight and obese have increased rapidly [6]. People who work long hours have irregular lifestyle and have little time to exercise, further promoting weight gain. Unbalanced consumption of foods high in energy and low in essential nutrients contributes to overweight or obesity among working-age adults, which has a further negative impact on health. If obesity is effectively controlled during this period, in the long run, obesity complications such as diabetes and stroke might be prevented, resulting in lower incidence of obesity complications at older ages and improved quality of life among the population.

Obesity is a complex metabolic disease that is related to multiple factors. It is believed to result from a combination of genetic, environmental, and social factors, among which consumption of an unhealthy diet plays an important role [7]. The current obesity epidemic is attributed to excessive energy intake and unbalanced energy intake. A high-quality diet is important for the prevention and treatment of a variety of chronic diseases including obesity, cancer, cardiovascular disease, and diabetes [8].

The Dietary Approaches to Stop Hypertension (DASH) and Mediterranean diets have been recognised as high quality diets worldwide. The DASH aimed to prevent and control high blood pressure by intaking reasonable diets. Studies have shown that the DASH diet has beneficial effects on obesity, hypertension, dyslipidemia, diabetes, gestational diabetes, and cardiovascular disease [9]. The Mediterranean diet is predominantly plant-based, rich in vegetables, fruit, nuts, legumes, fish or seafood, with low consumption of red meat and sweets and moderate intake of wine and dairy. Studies have proven that the Mediterranean diet can help to protect the heart and brain, prevent diabetes, prolong life, and reduce the risks of Parkinson disease, Alzheimer disease, and stroke [10]. One approach to assessing overall diet quality is to use a priori scores, based on dietary recommendations, such as DASH and alternate Mediterranean diet (aMed) scores. The quality of the DASH diet is quantified using DASH scores, and the aMed scores can reflect adherence to the Mediterranean diet. Many studies have confirmed that DASH and aMed scores reflect well the respective dietary quality [11].

Diets differ among different populations. Inner Mongolia is inland region, located in northwest China, and the population has dietary cultures and ethnic characteristics that differ from the rest of China. People in Inner Mongolia consume less vegetables and fruits, and more meat, oil and salt [12]. Inner Mongolia consists mainly of Han and Mongolian. Compared with Han population, the dietary pattern of Mongolians tends to be rich in whole milk, fats and oils, which might increase greater risk of obesity in Mongolia [13]. The prevalence of obesity in Inner Mongolia is relatively serious. Studies have shown that the prevalence of overweight and obesity in Inner Mongolia was 37.19 and 18.44% in 2014 [14], which was much higher than the national level [15] and higher than the global level [16]. Despite the heavy burden of obesity in the region, no current and comprehensive estimates of the obesity epidemic exist among working-age people in this part of China.

The main objective of this study was to assess the dietary quality of working-age people in Inner Mongolia using DASH and aMed scores. We also aimed to explore the relationship between dietary quality and obesity indicators using obesity as an entry point to provide a reasonable and healthy diet for the prevention of obesity, thereby reducing the incidence of obesity caused by dietary factors.

## Methods

### Study design and subjects

This cross-sectional study was a surveillance survey of chronic disease and nutrition in Chinese adults in Inner Mongolia conducted in 2015. The study design and questionnaire used have previously been described [12]. The surveillance survey consisted of dietary and non-dietary investigation, including information of sociodemographic characteristics, health behaviors, personal and familial medical history, and dietary information. Multi-stage cluster random sampling method was used to select a representative sample of residents aged 18 years and older. We selected the age range between 18 to 60 years as the working-age adults in this study.

### Diet data collection

The 24-h diet recall for 3 consecutive days and weighing method were used for the dietary investigation. The 24-h diet recall for 3 consecutive days, which is a dietary survey method recommended by the Chinese Dietary Guidelines, and aims to know about the residents’ intake of nutrients and foods. In the 24-h recall, every participant reported the type and amount of all foods (including alcohol) consumed in the past 24 h for 3 consecutive days. Information of condiments such as salt and soy sauce, and cooking oils consumption was collected with weighing method, in which changes in the household food inventory were measured. The average daily intake of nutrients and total energy were calculated according to the Chinese Food Composition Table, 2007 [17].

### Diet quality indices

DASH and aMed scores were used to assess the dietary quality of study participants. We calculated the DASH scores using the method described by Fung [18] et al. to determine adherence to the DASH diet, which consists of eight components. The scoring system is based on quintile rankings; for intakes of fruit; vegetables (excluding potatoes); low-fat dairy products; whole grains; and nuts, seeds, and legumes. Individuals receive a score from 1 (lowest quintile) to 5 (highest quintile). In contrast, individuals receive a score from 1 (highest quintile) to 5 (lowest quintile) for intakes of sodium, sugar-sweetened beverages, and red and processed meat. Male and female are classified into quintiles separately. A higher DASH score indicates greater compliance with the DASH diet, and therefore, a higher diet quality.

The aMed scores were developed by Fung [19] et al. to assess the quality of the Mediterranean diet. For foods and nutrients related to a beneficial health outcome, 1 point for each item is assigned to subjects with high intake of whole grains, vegetables, fruit, legumes, nuts, fish, and the ratio of monounsaturated to saturated fat, based on the sex-specific median of the study population; all other intakes receive 0 points. People with red and processed meat consumption below the median receive 1 point, and those whose consumption is at or above the median are assigned 0 points. For alcohol, 1 point is given for consumption between 5 and 25 g per day. The possible score range for aMed is 0 to 9, with higher scores indicating closer adherence to the Mediterranean diet.

### Anthropometric measurements

Height, weight, and waist circumference (WC) were directly measured by trained and evaluated health workers, using standard methods. Height and weight were measured once. WC was measured twice, and the mean value was used for the analyses.

### Definition of obesity

WC, BMI, and WC-BMI were used as indicators of obesity. Based on the World Health Organization recommendations for Asian adults, abdominal obesity was defined as WC ≥85 cm for female and ≥ 90 cm for male. BMI was calculated by dividing weight (kg) by the square of height (m^2^). According to the Chinese definitions [20], BMI was classified into two categories: underweight or normal weight (< 24 kg/ m^2^), and overweight and obese (≥24 kg/ m^2^). We combined WC and BMI as another weight-related risk outcome. BMI-WC is divided into low obesity risk and high obesity risk. Low risk individuals included those who had both BMI < 24 kg/ m^2^ and WC < 90 cm (male) or < 85 cm (female); high risk participants included those with BMI ≥24 kg/ m^2^ or WC ≥90 cm (male) or ≥ 85 cm (female).

### Other variables

Each participant completed a questionnaire about sociodemographic characteristics, health behaviors, and personal and familial medical history. Sociodemographic characteristics included gender, age, place of residence, ethnicity, and education level. Health behaviors included smoking status, drinking status, physical activity, and meals eaten outside the home. Personal and familial medical history include chronic diseases such as hypertension and diabetes mellitus.

### Statistical methods

Continuous variables following normal distribution were expressed as mean ± standard deviation (*SD*), and variables not following normal distribution were expressed as median (range, *P*_25_ to *P*_75_) and categorical variables were expressed as number (percentage). Participants were categorized based on tertiles of DASH scores and aMed scores. We used one-way analysis of variance or nonparametric test for continuous variables and chi-square test for categorical variables. Associations between diet quality and obesity indicators were examined using logistic regression models. In Model 1, we did not adjust for any factors; Model 2 was adjusted for sex, age, place of residence, ethnicity, education level, smoking status, drinking status, physical activity, meals eaten outside the home, family history of chronic diseases, hypertension, diabetes, and energy intake. A *P*-value < 0.05 (two-tailed) was used to indicate statistical significance. All statistical analyses were performed with IBM SPSS software version 19.0 (IBM Corp, Armonk, NY, USA).

## Results

### Study participant characteristics

The present study included 1320 working-age people aged 18 to 60 years. The characteristics of study participants across tertiles of diet quality indices are presented in Table 1. As shown in the table, most participants in the highest tertile of diet quality indices lived in urban, were Han ethnicity, and had higher education levels, sufficient physical activity, a family history of chronic diseases, and meals eaten outside the home (all *P* < 0.05).

**Table 1:** Characteristics of the subjects by DASH and aMed score groups^a^

		Total	DASH				aMed			
			T1	T2	T3	*P*	T1	T2	T3	*P*
WC	Abdominal obesity^a^	498(38.5)	184(40.9)	151(39.7)	163(35.1)	0.17	167(39.4)	194(42.7)	137(32.9)	0.01
BMI	Overweight and obesity^a^	784(60.6)	278(61.8)	236(62.1)	270(58.2)	0.42	276(65.1)	273(60.1)	235(56.5)	0.04
WC-BMI	High risk^a^	816(63.1)	286(63.6)	248(65.3)	282(60.8)	0.39	285(67.2)	286(6)	245(58.9)	0.04
Gender	Female	699(53.0)	228(49.9)	204(52.4)	267(56.3)	0.14	230(53.5)	233(50.5)	236(55.0)	0.40
Age	< 35	190(14.4)	51(11.1)	64(16.5)	75(15.8)	0.02	65(15.1)	56(12.2)	69(16.1)	0.002
	35–44	316(23.9)	130(28.5)	72(18.5)	114(24.1)		123(28.6)	95(20.6)	98(22.8)	
	45–54	535(40.5)	176(38.5)	168(43.2)	191(40.3)		160(37.2)	188(40.8)	187(43.6)	
	55–60	279(21.1)	100(21.9)	85(21.8)	94(19.8)		82(19.1)	122(26.4)	75(17.5)	
Place of residence	Urban	527(39.9)	122(26.7)	127(32.7)	278(58.7)	< 0.001	128(29.8)	151(32.8)	248(57.8)	< 0.001
Ethnicity	Han	1034(78.3)	322(70.5)	308(79.2)	404(85.2)	< 0.001	321(74.6)	362(78.5)	351(81.8)	< 0.001
	Mongolian	233(17.7)	123(26.9)	65(16.7)	45(9.5)		97(22.6)	88(19.1)	48(11.2)	
	Other minority	53(4.0)	12(2.6)	16(4.1)	25(5.3)		12(2.8)	11(2.4)	30(7.0)	
Education level	Low	476(36.1)	227(49.7)	140(36.0)	109(23.0)	< 0.001	201(46.7)	184(39.9)	91(21.2)	< 0.001
	Medium	485(36.7)	157(34.3)	147(37.8)	181(38.2)		149(34.7)	162(35.1)	174(40.6)	
	High	359(27.2)	73(16.0)	102(26.2)	184(38.8)		80(18.6)	115(25.0)	164(38.2)	
Smoking status	Current smokeing	409(31.0)	157(34.4)	117(30.1)	135(28.5)	0.40	141(32.8)	150(32.6)	118(27.5)	0.33
	Quitting smoking	55(4.2)	17(3.7)	17(4.4)	21(4.4)		15(3.5)	18(3.9)	22(5.1)	
	not smoking	856(64.8)	283(61.9)	255(65.5)	318(67.1)		274(63.7)	293(63.5)	289(67.4)	
Physical activity	None	100(7.6)	43(9.4)	38(9.8)	19(4.0)	< 0.001	37(8.6)	41(8.9)	22(5.1)	0.001
	Inadequate	376(28.5)	99(21.7)	113(29.0)	164(34.6)		110(25.6)	112(24.3)	154(35.9)	
	Suffıcient	844(63.9)	315(68.9)	238(61.2)	291(61.4)		283(65.8)	308(66.8)	253(59.0)	
Meals eaten outside the home	Yes	230(17.4)	62(13.6)	78(20.1)	90(19.0)	0.03	58(13.5)	90(19.5)	82(19.1)	0.03
Drinking status	Never	802(60.8)	275(60.2)	240(61.7)	287(60.6)	0.12	270(62.8)	266(57.7)	266(62.0)	0.18
	Moderate	367(27.8)	126(27.6)	96(24.7)	145(30.6)		119(27.7)	129(28.0)	119(27.7)	
	Excess	151(11.4)	56(12.2)	53(13.6)	42(8.6)		41(9.5)	66(14.3)	44(10.3)	
Family history	Yes	771(58.4)	243(53.2)	222(57.1)	306(64.6)	0.002	224(52.1)	261(56.6)	286(66.7)	< 0.001
Hypertension	Yes	552(41.8)	195(42.7)	169(43.4)	188(39.7)	0.48	173(40.2)	194(42.1)	185(43.1)	0.69
Diabetes	Yes	84(6.4)	26(5.7)	25(6.4)	33(7.0)	0.73	24(5.6)	36(7.8)	24(5.6)	0.29

Variables are presented as frequency (percentage) (%)

^a^After standardization using the 2010 National Census Data, the standardized prevalence rates of abdominal obesity, overweight obesity, and high risk of obesity were 34.5, 56.8, and 58.6%, respectively

### Outcome data

In total, 498 (38.5%) participants had abdominal obesity, 784 (60.6%) were overweight and obesity, and 816 (63.1%) were at high risk of obesity. After standardization using 2010 National Census Data, the standardized prevalence rates of abdominal obesity, overweight and obesity, and high risk of obesity were 34.5, 56.8, and 58.6%, respectively (Table 1).

### Food group and nutrients intakes

Compared with participants who had lower DASH scores, those with higher DASH scores had higher intakes of all foods except alcohol, red meat and processed meat, and sweetened beverages, as well as lower intakes of fat, total fatty acids, saturated fatty acids, monounsaturated fatty acids, and sodium (*P*_trend_ < 0.001). Individuals in the third tertile of aMed scores had significantly higher food intakes except for alcohol, red and processed meat, low-fat dairy, and sweetened beverages (*P*_trend_ < 0.001), as compared with participants in the first tertile (Table 2).

**Table 2:** Dietary and nutrients intakes according to tertile of diet score

		DASH				aMed			
	Total	T1	T2	T3	*P*_*trend*_	T1	T2	T3	*P*_*trend*_
Food groups (g/day)									
Vegetables	114.3 ± 100.7	69.4 ± 74.5	110.8 ± 87.7	160.57 ± 111.83	< 0.001	67.9 ± 71.5	115.1 ± 95.9	160.2 ± 109.4	< 0.001
Fruits	40.4 ± 76.2	8.4 ± 26.2	27.3 ± 62.0	82.1 ± 97.45	< 0.001	12.7 ± 31.8	29.9 ± 67.5	79.5 ± 97.8	< 0.001
Nuts	2.1 ± 9.4	1.2 ± 6.00	1.1 ± 5.3	3.73 ± 13.55	< 0.001	1.4 ± 6.2	2.2 ± 11.1	2.7 ± 10.0	0.04
Whole grains	19.2 ± 33.9	8.7 ± 23.4	21.0 ± 34.4	27.75 ± 38.88	< 0.001	10.7 ± 26.4	18.1 ± 34.7	28.7 ± 37.2	< 0.001
Legumes	26.2 ± 51.8	7.5 ± 20.5	18.8 ± 43.7	50.28 ± 67.5	< 0.001	22.9 ± 55.0	23.9 ± 48.5	32.0 ± 51.6	0.01
Fish	7.5 ± 26.8	3.7 ± 16.4	6.0 ± 18.9	12.31 ± 37.59	< 0.001	3.9 ± 16.0	8.0 ± 30.4	10.5 ± 30.7	< 0.001
Red and processed meats	75.9 ± 90.0	107.7 ± 112.9	62.4 ± 68.1	56.52 ± 70.71	< 0.001	120.4 ± 115.0	65.7 ± 72.5	42.5 ± 53.2	< 0.001
Alcohol	12.3 ± 69.6	10.1 ± 64.7	19.4 ± 94.6	8.73 ± 45.73	0.77	8.0 ± 59.7	14.1 ± 74.3	14.8 ± 73.6	0.16
Low-fat dairy	2.1 ± 12.8	1.4 ± 6.1	1.4 ± 6.3	3.38 ± 19.6	0.02	2.2 ± 8.7	1.2 ± 5.6	3.0 ± 19.8	0.34
Sweetened beverages	0.4 ± 7.1	0.6 ± 3.1	1.1 ± 12.7	0.01 ± 0.15	0.77	0.2 ± 3.2	0.2 ± 3.9	0.8 ± 11.4	0.20
Nutrients									
Energy (Kcal)	1689.2 ± 750.7	1736.9 ± 811.4	1590.8 ± 703.6	1724.07 ± 720.91	0.79	1769.3 ± 837.9	1615.4 ± 706.5	1688.2 ± 695.8	0.11
Fat (g/day)	66.2 ± 48.7	76.5 ± 57.7	61.4 ± 50.4	60.84 ± 34.36	< 0.001	75.3 ± 57.3	63.3 ± 46.0	60.8 ± 40.5	< 0.001
Carbohydrate (g/day)	225.0 ± 105.2	211.8 ± 95.5	215.9 ± 98.0	245.28 ± 116.36	< 0.001	217.9 ± 101.4	216.2 ± 95.44	241.7 ± 116.6	0.001
Protein (g/day)	51.2 ± 26.2	52.3 ± 28.4	47.0 ± 21.1	53.67 ± 27.43	0.43	57.3 ± 30.1	47.96 ± 23.4	48.6 ± 23.9	< 0.001
Cholesterol (mg/day)	210.1 ± 194.5	209.2 ± 185.0	210.8 ± 185.3	210.49 ± 210.59	0.92	235.5 ± 194.2	188.3 ± 165.9	208.2 ± 219.3	0.04
Dietary fiber (g/day)	7.5 ± 4.7	5.7 ± 3.4	6.8 ± 4.0	9.82 ± 5.24	< 0.001	6.1 ± 3.9	7.1 ± 4.6	9.4 ± 4.8	< 0.001
Total fatty acid (g/day)	31.7 ± 22.9	36.3 ± 25.5	27.8 ± 20.6	30.74 ± 21.51	< 0.001	37.1 ± 25.2	29.9 ± 22.5	28.5 ± 20.0	< 0.001
Saturated fatty acid(g/day)	10.3 ± 8.5	12.7 ± 10.2	9.1 ± 7.6	9.23 ± 7.07	< 0.001	13.8 ± 10.1	9.6 ± 7.6	7.8 ± 6.5	< 0.001
Monounsaturated fatty acid (g/day)	12.5 ± 9.9	14.8 ± 11.5	11.0 ± 8.9	11.7 ± 8.78	< 0.001	15.0 ± 11.2	11.8 ± 9.7	10.8 ± 8.3	< 0.001
Polyunsaturated fatty acid (g/day)	8.2 ± 6.5	8.0 ± 5.5	7.2 ± 6.2	9.24 ± 7.29	< 0.001	7.5 ± 5.2	7.9 ± 6.9	9.3 ± 7.0	< 0.001
Potassium (g/day)	1.3 ± 0.7	1.2 ± 0.7	1.7 ± 0.6	1.42 ± 0.69	< 0.001	1.4 ± 0.8	1.2 ± 0.6	1.3 ± 0.6	0.37
Sodium (g/day)	5.2 ± 4.3	6.3 ± 4.8	5.7 ± 4.9	3.72 ± 2.36	< 0.001	5.1 ± 4.4	5.3 ± 4.1	5.2 ± 4.4	0.61

Values are presented as *mean* ± *SD*

*P* and *P*_trend_ obtained from ANOVA

### Association between diet quality scores and obesity indicators

In univariate analyses, there was no significant association between dietary quality, as assessed using DASH scores, and obesity indicators. After adjustment for confounding factors, higher diet quality assessed using DASH was only associated with WC. The odds ratio (*OR*) for abdominal obesity in the highest tertile of DASH scores, compared with the lowest, was 0.71 (95% confidence interval (*CI*) 0.53, 0.96; *P*_trend_ = 0.03). Furthermore, aMed was inversely associated with obesity indicators. After adjusting for confounding factors, OR for abdominal obesity in the highest tertile of a aMed scores, compared with the lowest were 0.63 (95% *CI* 0.47, 0.87; *P*_trend_ = 0.005) and 0.57 (95% CI 0.41, 0.77; *P*_trend_ = 0.02) for overweight and obesity, respectively, and 0.60 (95% *CI* 0.44, 0.81; *P*_trend_ = 0.02) for high obesity risk. Confounding factors included gender, age, place of residence, ethnicity, education level, smoking status, drinking status, physical activity, meals eaten outside the home, family history of chronic diseases, hypertension, diabetes, and energy intake, which may influence the results when assessing the relationship between diet and obesity indicators (Table 3).

**Table 3:** Logistics regression model of diet quality scores and obesity indicators

		T1	T2	T3	*P*	*P*_*trend*_
			OR (95% CI)	OR (95% CI)		
DASH						
WC	Model1	1.00	0.95(0.72,1.26)	0.78(0.60,1.02)	0.07	0.13
	Model2	1.00	0.95(0.70,1.27)	0.71(0.53,0.96)	0.03	0.03
BMI	Model1	1.00	1.01(0.77,1.34)	0.86(0.66,1.12)	0.27	0.27
	Model2	1.00	0.99(0.74,1.33)	0.76(0.57,1.02)	0.07	0.07
WC-BMI	Model1	1.00	1.08(0.81,1.43)	0.89(0.68,1.16)	0.39	0.38
	Model2	1.00	1.06(0.79,1.43)	0.82(0.61,1.09)	0.17	0.12
aMed						
WC	Model1	1.00	1.15(0.88,1.50)	0.76(0.57,1.00)	0.05	0.04
	Model2	1.00	1.10(0.83,1.46)	0.64(0.47,0.87)	0.004	0.005
BMI	Model1	1.00	0.81(0.62,1.06)	0.70(0.53,0.92)	0.01	0.19
	Model2	1.00	0.76(0.57,1.02)	0.57(0.41,0.77)	< 0.001	0.02
WC-BMI	Model1	1.00	0.83(0.63,1.10)	0.70(0.53,0.93)	0.01	0.10
	Model2	1.00	0.79(0.59,1.06)	0.60(0.44,0.81)	0.001	0.02

Model 1: crude data;

Model 2: Adjusted for gender, age, place of residence, ethnicity, education level, smoking status, drinking status, physical activity, meals eaten outside the home, family history of chronic diseases, hypertension, diabetes and energy intake.

## Discussion

In this cross-sectional study, we evaluated the diet quality of the working-age adults in Inner Mongolia in 2015 and its association with obesity indicators. Overweight and obesity are common among the working-age adults. The standardized prevalence of abdominal obesity, overweight and obesity, and high obesity risk were 34.5, 56.8, and 58.6%, respectively. In the same year, the rate of overweight and obesity among Chinese adults over 18 years old was 42.6% [21]. The prevalence of overweight and obesity was 38.6 and 18.4%, respectively, among the working-age adults in Spain [22]. As a developing country, the prevalence of overweight among Malaysian adults was 51.2% [23]. In 2014, the prevalence rate of overweight and obesity in 20 European countries reached 53.1% [24].

Some studies [25, 26] in the Mediterranean region about Mediterranean Diet are using MEDAS to determine the level of adherence to the Mediterranean diet. However, MEDAS is not very suitable for the non-Mediterranean region. Therefore, we used DASH and aMed scores to assess the dietary quality of working-age adults in Inner Mongolia. The overall dietary quality score of the working-age adults in Inner Mongolia was lower and the dietary quality was poor. The median aMed score for participants was only 3(range, 2 to 4), and the median DASH score was 21(range, 19 to 24) (Table S1). A case-control study in China showed that the DASH score of residents in Hong Kong was 22.6 [27]; another case-control study showed that the aMed score for residents of Guangzhou, China was 3.92 [28]. To our knowledge, this is the first observational study investigating the association between obesity indicators and diet quality, as assessed by DASH and aMed, among the working-age adults of Inner Mongolia. A meta-analysis showed that the Mediterranean diet was associated with a reduced risk of metabolic syndrome and all components, including abdominal obesity [29]. In a randomized controlled clinical trial, after 3 months of dietary intervention, Azadi-Yazdi found that the participants following DASH diet had reduced BMI [30]. The results of an interventional study showed that adherence to the Mediterranean diet was associated with lower body weight and lower waist circumference [31]. Consistent with other findings [26–28], our study showed that the DASH score was inversely associated with WC whereas the aMed dietary score was negatively correlated with WC, BMI, and WC-BMI. These results indicated that the Mediterranean diet is more closely related to obesity compared with the DASH diet in our population.

The intake of red and processed meats among our participants with higher aMed scores was much lower than in those with higher DASH scores. As an important part of both the DASH diets and Mediterranean diets, red and processed meats are rich in protein, cholesterol, and saturated fatty acids, which provide greater energy; there is a significant positive correlation between energy intake and obesity [32]. Epidemiological studies have indicated that the consumption of red meat is positively correlated with general and abdominal obesity [33]. According to reports, adhering to a meat diet model for 5 years increases the risk of general obesity [34]. A national cross-sectional study in the United States reported that those who consumed more meat had a much higher daily total energy intake, and red meat was positively associated with central obesity [35]. Furthermore, a study conducted in the Chinese population showed that intake of total red meat, intake of fresh red meat, and intake of fatty fresh red meat were positively associated with an increased WC measurement (men only) and risk of abdominal obesity among Chinese adults aged 18 to 75 [36]. These studies suggest the role that red and processed meats plays in obesity.

Working-age participants with a higher-quality diet, as assessed using aMed scores, had lower energy intake than those with high dietary quality, assessed using DASH scores. The fundamental cause of obesity is an energy imbalance between calories consumed and calories expended [37]. Epidemiological studies in Europe and the United States have found a significant positive correlation between energy intake and obesity-related indicators such as weight gain, BMI, and WC [38]. A recent meta-analysis showed that limiting the intake of energy can promote weight loss and improve metabolism [39].

People with higher dietary quality assessed by DASH had higher consumption of total fatty acids and saturated fatty acids than those with higher dietary quality assessed by aMed. However, a growing body of evidence suggests that the level of saturated fatty acid intake is positively correlated with the occurrence of cardiovascular and cerebrovascular diseases [40]. A 4-week experimental study among overweight or obese men showed that substituting dietary saturated fat with unsaturated fat can induce a significant loss of body weight and fat mass without a significant change in total energy or fat intake [41]. Saturated fatty acids in the diet can reduce leptin deficiency or leptin resistance and induce body obesity [42]. This may also be the reason why aMed was more closely related to obesity indicators than DASH in our population.

The DASH considers the component of sweetened beverages separately, with a recommendation of five servings or less per week [43]. Lower intake will affect the dietary quality of DASH evaluation. However, consumption of sweetened beverages in our population was very low, with average daily intake only 0.37 g, which is far below the standard intake. And sweetened beverage is not one of items of aMed. Therefore, sweetened beverages are a factor that affects the relationship between diet quality and obesity. It is well known that sweetened beverages are harmful to metabolism. A recent study published in *Diabetes Care* found that body weight increased more among those who increased their sugary beverage consumption than among those who maintained a stable consumption or decreased their consumption of sweetened beverages [44]. Sweetened beverages have a lower degree of satiety than solid foods containing the same amount of calories and their consumption stimulates appetite, which may lead to greater calorie intake [45]. Therefore, when using DASH to assess the dietary quality among our population, DASH scores only reflected the sweetened beverage intake of some people, which represents only a small part of the cause of obesity. This may be the reason why aMed is more suitable for evaluating the relationship between obesity and diet quality than DASH.

In our study, cholesterol intake gradually decreased with higher diet quality, according to the Mediterranean diet. Interestingly, compared with higher aMed scores, the intake of cholesterol was higher in participants with higher DASH scores, and the intake of cholesterol increased gradually with increased dietary quality. A cohort study published in *JAMA* recently showed that higher consumption of dietary cholesterol was significantly associated with higher risk of incident cardiovascular disease and all-cause mortality [46]. Therefore, our results showed that aMed is more able to reflect the quality of the diet, it is suitable for evaluating the dietary quality of the Inner Mongolian population compared with DASH.

After analyzing the relationship between food components and obesity indicators according to the DASH and aMed indices, we found that people with high quality scores in the DASH diet and Mediterranean diet had relatively high scores for fruits, vegetables, whole grains, nuts and legumes, and red and processed meats. These results indicate that the composition of these two dietary patterns have many similarities. Moreover, the DASH and Mediterranean diets also differ in many respects. DASH is known as Dietary Approaches to Stop Hypertension, which considers sodium intake separately. A large number of studies have shown that potassium and sodium are associated with hypertension, the prognosis of chronic kidney disease, and cardiovascular events. Compared with participants who had higher dietary quality according to aMed, the intake of potassium was higher and sodium was lower in those with higher DASH dietary quality scores in our study. So comprehensively, DASH is suitable for assessing the relationship between dietary quality and certain diseases such as hypertension, and aMed is more suitable for assessing the relationship between dietary quality and obesity.

In addition to being a tool for preventing obesity, growing evidence shows that the Mediterranean diet is associated with diabetes, hypertensive, metabolic syndrome and other diseases [47]. The Mediterranean diet demonstrated to exert a preventive effect toward cardiovascular diseases, in both Mediterranean and non-Mediterranean populations [47, 48]. Some studies also suggested a potential role in preventing certain cancers [49]. Newer research has showed that higher adherence to the Mediterranean diet was associated with lower risk of cognitive decline, depression, and other mental disorders [50]. Therefore, the Mediterranean diet could be used in public health nutrition policies in order to promote health.

### Limitations

There are several limitations to this study. First, the study design was cross-sectional, the associations are not proof of causality, and reverse causality bias could be present. Additional cohort studies with follow-up data are necessary to strengthen the understanding of the associations identified here. Secondly, our research was conducted in Inner Mongolia, which investigated the local dietary and evaluated the relationship between dietary quality and obesity. Different from other parts of China, the diet in Inner Mongolia residents is characterized by less intake of vegetables, fruits and fish, and more intake of meat. Our conclusion may be applicable to those regions whose diet characteristics are similar with diets in Inner Mongolia. At the same time, our research population was obtained by the Multi-stage cluster random sampling method, and the samples are well representative. Thirdly, a 24-h recall and weighing method were used for the dietary investigation, which may cause recall bias. Considering the limitations of study, our results need to be confirmed in further studies, especially for prospective studies in Inner Mongolia.

### Strengths

Our study population chose working age population. If obesity is effectively controlled in the working age population, it can prevent diseases such as diabetes and stroke, thereby reducing the incidence of obesity complications in the elderly and improving people’s quality of life. We evaluated the relationship between diet and obesity, and finally found that aMed is more suitable for evaluating the relationship between diet quality and obesity in Inner Mongolia, and we can prevent obesity by the Mediterranean diet.

## Conclusions

A higher diet quality, as assessed using aMed scores, was more closely associated with obesity among working-age people in Inner Mongolia as compared with assessment using DASH scores. The Mediterranean diet can be recommended for underdeveloped regions as a healthy diet to control weight.

## Supplementary information

**Additional file 1: Table S1.** Characteristics Total and component scores of the diet quality indices

## Data Availability

The full data set are available from the corresponding author under reasonable request.

## Abbreviations

95% *CI*: 95% Confidence interval
BMI: Body mass index
*OR*: Odds ratio
DASH: The Dietary Approaches to Stop Hypertension:
aMed: Alternate Mediterranean diet
WC: Waist circumference
*SD*: Standard deviation
