# Supplementary material for: Association between diet quality and obesity indicators among the working-age adults in Inner Mongolia, Northern China: a cross-sectional study
Source: BMC Public Health. 2020 Jul 25;20:1165. doi: 10.1186/s12889-020-09281-5 (PMC7382798; doi:10.1186/s12889-020-09281-5)
Supplement: Supplementary file 1 — Additional file 1: Table S1. Characteristics Total and component scores of the diet quality indices [file 12889_2020_9281_MOESM1_ESM.docx]

| **Table S1.** Characteristics Total and component scores of the diet quality indices | | | | | |
| --- | --- | --- | --- | --- | --- |
|  | T1 | T2 | T3 | total | *P* |
| DASH |  |  |  |  |  |
| Total score (8-40) | 18(16,19) | 19(18,21) | 23(21,26) | 21(19,24) | <0.001 |
| Component scores | |  |  |  |  |
| Vegetables (1-5) | 2(1,3) | 3(2,4) | 4(2,5) | 3(2,4) | <0.001 |
| Fruits (1-5) | 1(1,1) | 1(1,1) | 2(1,4) | 1(1,3) | <0.001 |
| Whole grains (1-5) | 1(1,1) | 1(1,3) | 2(1,4) | 2(1,3) | <0.001 |
| Low-fat dairy (1-5) | 1(1,1) | 1(1,1) | 1(1,1) | 1(1,1) | 0.001 |
| Nuts and legumes (1-5) | 1(1,1) | 1(1,2) | 2(1,4) | 1(1,3) | <0.001 |
| Red and processed meat (1-5) | 2(1,3) | 3(2,4) | 3(2,5) | 3(2,4) | <0.001 |
| Sweetened beverages (1-5) | 5(5,5) | 5(5,5) | 5(5,5) | 5(5,5) | 0.058 |
| Sodium (1-5) | 3(2,4) | 3(2,4) | 3(2,4) | 3(2,4) | 0.572 |
| aMed |  |  |  |  |  |
| Total score (0-9) | 1(1,1) | 2(2,2) | 4(3,5) | 3(2,4) | <0.001 |
| Component scores | |  |  |  |  |
| Vegetables (0-1) | 0(0,0) | 0(0,1) | 1(0,1) | 1(0,1) | <0.001 |
| Fruits (0-1) | 0(0,0) | 0(0,0) | 1(0,1) | 0(0,1) | <0.001 |
| Nuts (0-1) | 0(0,0) | 0(0,0) | 0(0,0) | 0(0,0) | <0.001 |
| Whole grains (0-1) | 0(0,0) | 0(0,1) | 1(0,1) | 0(0,1) | <0.001 |
| Legumes (0-1) | 0(0,0) | 0(0,0) | 1(0,1) | 0(0,1) | <0.001 |
| Fish (0-1) | 0(0,0) | 0(0,0) | 0(0,0) | 0(0,0) | <0.001 |
| Ratio of monounsaturated to saturated fat (0-1) | 0(0,0) | 0(0,1) | 1(0,1) | 0.5(0,1) | <0.001 |
| Red and processed meats (0-1) | 0(0,0) | 0(0,1) | 1(0,1) | 0(0,1) | <0.001 |
| Alcohol (0-1) | 0(0,0) | 0(0,0) | 0(0,0) | 0(0,0) | 0.074 |
| Variables are presented as median (*p*_25_-*p*_75_) | | | | | |
